# Expression and significance of angiopoietin-2 and cyclin D1 in laryngeal squamous cell carcinoma and the correlation with prognosis

**DOI:** 10.3892/etm.2013.1282

**Published:** 2013-09-02

**Authors:** YI-FEI LIU, JIAN-GUO ZHANG, HAO-SHENG NI, HAO LIU, SHU ZHANG, HUA HUANG, GONG-SHENG SHI

**Affiliations:** 1 Departments of Pathology, Affiliated Hospital of Nantong University, Nantong, Jiangsu 226001, P.R. China; 2 Otolaryngology, Affiliated Hospital of Nantong University, Nantong, Jiangsu 226001, P.R. China

**Keywords:** laryngeal squamous cell carcinoma, angiopoietin-2, cyclin D1, prognosis

## Abstract

The aim of this study was to study the expression of angiopoietin-2 (Ang-2) and the cell cycle protein D1 (cyclin D1) in laryngeal squamous cell carcinoma (SCC), and its clinicopathological meaning. The expression of Ang-2 and cyclin D1 was analyzed in 116 cases of laryngeal SCC, five cases of atypical hyperplasia and five cases of vocal polyp tissues using the immunohistochemical streptavidin-perosidase (S-P) method. Ang-2 and cyclin D1 protein expression was not present in 5 cases of atypical hyperplasia or 5 cases of vocal cord polyps. However, the proportions of positive staining in well, moderately and poorly differentiated laryngeal SCC were 40, 66.7 and 100%, respectively, for Ang-2 and 50, 66.7, 100%, respectively, for cyclin D1, and were statistically significant (P<0.05). The expression of Ang-2 was positively correlated with cyclin D1 in the laryngeal SCC (r=0.5042; P<0.05). This showed that the tumor grading and cyclin D1 were independent factors affecting laryngeal SCC patient survival by the Cox regression model of risk factors proportion analysis. Ang-2 is synergistic with cyclin D1 in the development of laryngeal SCC. Ang-2 is associated with the metastasis of lymph nodes. Detection of both Ang-2 and cyclin D1 may possess clinical significance in evaluating the prognosis and guiding the clinical treatment of SCC.

## Introduction

In China, laryngeal squamous cell carcinoma (SCC) accounts for ~7.9–35% of all the head and neck malignant tumors, and ~1.2–1.6% of all malignant tumors (1). The induction and development of tumors involves multiple factors (2). As the tumor grows larger than 1 × 1 × 2 mm, its further expansion relies on the growth of new inner blood vessels, which is subject to the modulation of various cytokines. Angiopoietin (Ang) and its receptor Tie-2 are the two key cytokines regulating the development of tumor blood vessels (3). The positive rate of Ang-2 expression in laryngeal SCC is significantly higher than in normal mucosa tissue. As the differentiation decreased, the expression of Ang-2 increased The overexpression of Ang-2 contributes to the occurrence of laryngeal SCC. It appears that laryngeal SCC is associated with the expression of Ang-2. Previous studies show that cyclin D1 is not or seldom expressed in the normal tissue, but it is highly expressed in numerous malignant tumors. Cyclin D1 is associated with the tumor classification and may be regarded as a potential prognostic indicator. The aim of this study is to investigate the expression of Ang-2 and cyclin D1 in the laryngeal SCC and clinicopathological signification. It is likely that they play a significant role in tumor induction, development and metastasis (4).

## Materials and methods

### Materials

A total of 116 laryngeal SCC specimens were collected between January 2003 and December 2011 from The Otolaryngology Department of The Affiliated Hospital of Nantong University (Nantong, China). The complete clinical and pathological materials were well preserved. Of these patients, there were 107 males and 9 females aged from 42 to 83 years, with an average age of 62.8 years. According to the standard classification of head and neck neoplasm histopathology listed by the WHO (World Health Organization) (5) in 2005, 60 cases were well differentiated, 45 moderately differentiated and 11 poorly differentiated. The patients studied were initial cases without radiotherapy, chemotherapy or biotherapy before surgery. In addition, five cases of vocal cord polyp and five cases of atypical hyperplasia were used as control groups. We tracked the survival condition of 116 cases following surgery and recorded the causes of mortality in patients. For example, certain individuals succumbed to tumor, accident or other causes. The calculation of overall survival (OS) starts from the established diagnosis to the mortality caused by the tumor or the termination of follow-up study. Any mortality irrelevant to laryngeal SCC was recorded as truncation. This study was approved by the Ethics Committee of Affiliated Hospital of Nantong University, Nantong, China). Written informed consent was obtained from all patients.

### Specimen preparation

Fresh tissues were fixed with 4% paraformaldehyde, embedded with paraffin, cut into serial sections with a thickness of 4 *μ*m and stained with hematoxylin and eosin and Ang-2 and cyclin D1 immunohistochemical stains.

### Immunohistochemical staining

Paraffin sections were deparaffinized, dehydrated and antigen retrieval was conducted by microwave for 15 min within citrate buffer solution (pH 6.0). Droplets of polyclonal rabbit anti-human Ang-2 and cyclin D1 were distributed into the deparaffinized sections for streptavidin-perosidase (S-P) staining. The known positive section was used as a positive control, normal goat serum as a negative control in place of primary antibody and PBS as a blank control also in place of primary antibody.

### Immunohistochemical scoring

Ang-2 was located in the cytoplasm of tumor cells. Positive staining is observed as bright yellow, brown yellow or brown granules focally or diffusively distributed. Cyclin D1 was located in the tumor cell nucleus. Cyclin D1 protein expression is indicated by bright yellow, brown yellow or brown granules focally or diffusively distributed. Semi-quantitative results were obtained under a microscope (Olympus BX51-P; Olympus, Tokyo, Japan) using the method of Xu and Yang (6), which is dependent on the staining intensity and number of positive cells. Standard scores of staining intensity were as follows: no staining, 0 points; bright yellow, 1 point; brown yellow, 2 points; brown, 3 points. Number of positive cells under the same objective: negative, 0; number of stained cells within one field of vision ≤10%, 1 point; 11–50%, 2 points; 51–75%, 3 points; ≥76%, 4 points. The result of the two scores multiplied was graded: 0–2 as (-); 3 as (+); 4 as (++); ≥5 as (+++); results between + and +++ indicate to presence of expression.

### Statistical analysis

SPSS 17.0 (SPSS, Inc., Chicago, IL, USA) was employed to statistically process the relevant data. The χ^2^ test was used to compare the expression of Ang-2 and cyclin D1 in each group. The correlation comparison between Ang-2 and cyclin D1 was calculated using the Spearman’s rank correlation analysis. P<0.05 was considered to indicate a statistically significant result.

## Results

### Expression of Ang-2 in vocal cord polyp, atypical hyperplasia and laryngeal SCC tissues

**Figure 1.**
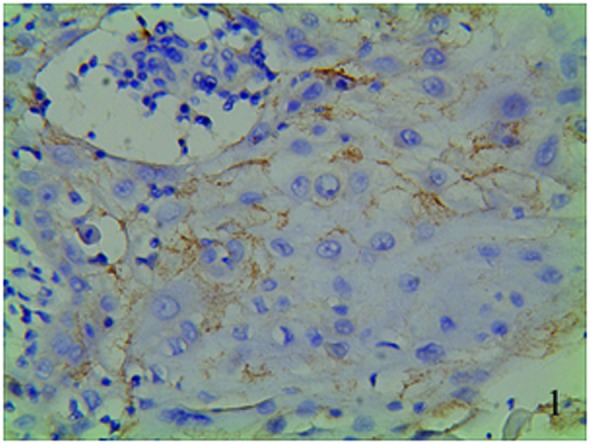
Expression of Ang-2 in well differentiated laryngeal SCC: the cytoplasm of tumor cells presents bright yellow (magnification ×400). Ang-2, angiopoietin-2; SCC, squamous cell carcinoma.

**Figure 2.**
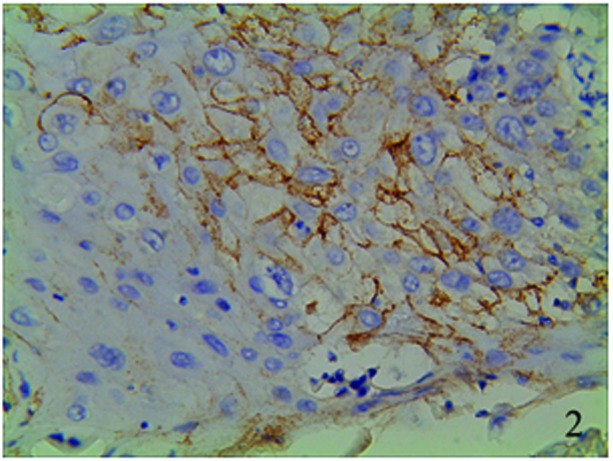
Expression of Ang-2 in moderately differentiated laryngeal SCC: the cytoplasm of tumor cells presents as brown yellow (magnification ×400). Ang-2, angiopoietin-2; SCC, squamous cell carcinoma.

**Figure 3.**
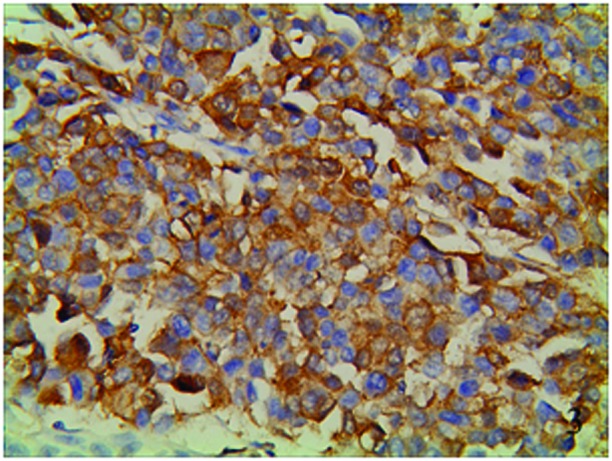
Expression of Ang-2 in poorly differentiated laryngeal SCC: the cytoplasm of tumor cells presents as brown (magnification ×400). Ang-2, angiopoietin-2; SCC, squamous cell carcinoma.

**Figure 4.**
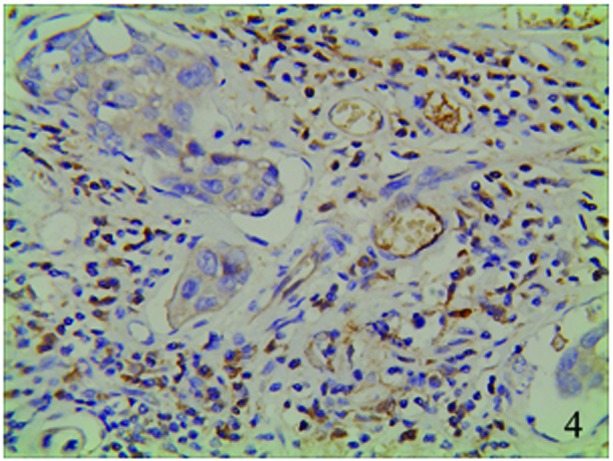
Expression of Ang-2 in moderately differentiated laryngeal SCC: the cytoplasm of tumor cells presents as brown yellow while the vascular endothelial cells are brown (magnification ×400). Ang-2, angiopoietin-2; SCC, squamous cell carcinoma.

The positive rate of Ang-2 was 0% (0/5) in vocal cord polyp and 0% (0/5) in atypical hyperplasia. The positive rate in laryngeal SCC tissues was 40% for well differentiated, 66.7% for moderately differentiated and 100% for poorly differentiated. The positive staining located in the cytoplasm was as bright yellow, brown yellow or brown granules focally or diffusively distributed (Figs. 1–3). Ang-2 expression presented as brown staining in the vascular endothelial cells (Fig. 4). one group from the groups of the vocal cord polyp and atypical hyperplasia and one group from the groups of well, moderately and poorly differentiated laryngeal SCC, statistical significance was indicated (P<0.05). A pairwise comparison was conducted among the well, moderately and poorly differentiated laryngeal SCC groups (P<0.05; Table I).

### Expression of cyclin D1 in vocal cord polyp, atypical hyperplasia and laryngeal SCC tissues

**Figure 5.**
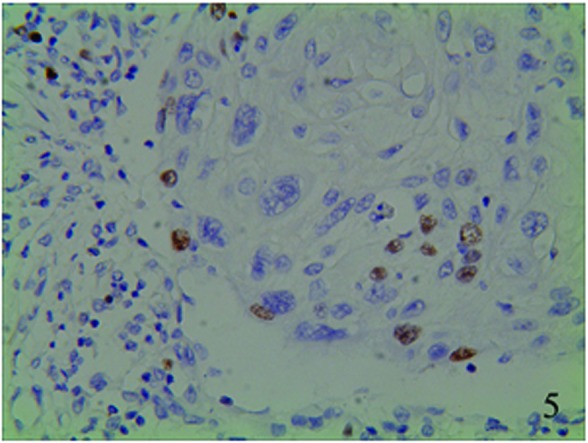
Expression of cyclin D1 in well differentiated laryngeal SCC: the nucleus of tumor cells presents as bright yellow (magnification ×400). SCC, squamous cell carcinoma.

**Figure 6.**
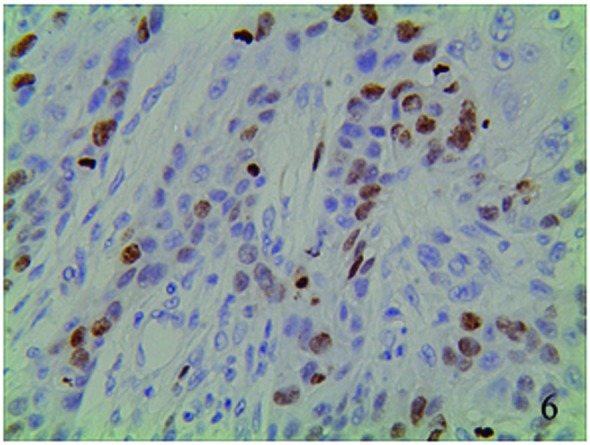
Expression of cyclin D1 in moderately differentiated laryngeal SCC: the nucleus of tumor cells presents as brown yellow (magnification ×400). SCC, squamous cell carcinoma.

**Figure 7.**
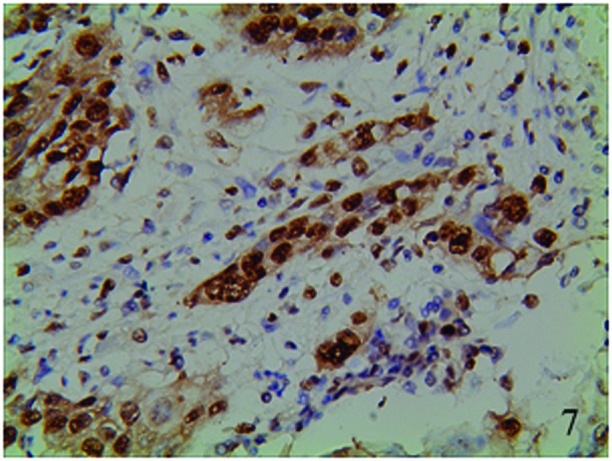
Expression of cyclin D1 in poorly differentiated laryngeal SCC: the nucleus of tumor cells suffuses as brown (magnification ×400). SCC, squamous cell carcinoma.

**Figure 8.**
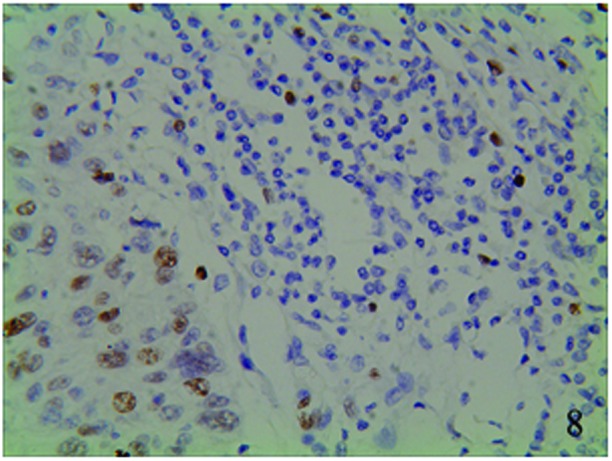
Expression of cyclin D1 in the tumor cell infiltrating into lymph gland: the nucleus of tumor cells presents as brown (magnification ×400).

The incidence of cyclin D1 expression was 0% (0/5) in vocal cord polyp and 0% (0/5) in atypical hyperplasia. As for its positive rate in laryngeal SCC tissues, it was 50% for well differentiated, 66.7% for moderately differentiated and 100% for poorly differentiated laryngeal SCC. The positive staining was located in the nucleus as bright yellow, brown yellow or brown granules (Figs. 5–7). The staining was focally positive and infiltrated into the lymph gland (Fig. 8). The interclass differences between the well, moderately and poorly differentiated laryngeal SCC groups and the vocal cord polyp and atypical hyperplasia groups was statistically significant (P<0.05). Interclass expressions for the well and moderately differentiated groups and the well and poorly differentiated groups were statistically significant (P<0.05). No statistical significance was identified between the moderately and poorly differentiated groups (Table II).

### Association between Ang-2 expression in laryngeal SCC and clinically pathological factors

The proportions of positive staining of Ang-2 in the well, moderately and poorly differentiated laryngeal SCC groups were 40, 66.7 and 100%, respectively. Differences exist among the three groups, for example, the poorly differentiated group showed an expression rate higher than those of the well and moderately differentiated groups. The interclass difference was statistically significant (Z=4.020; P<0.05). The proportions of positive staining of laryngeal SCC in TNM grades T1+T2 and T3+T4 were 46.7 and 73.2%, respectively. The positive rate of Ang-2 increases at later pathological stages. The Ang-2 expression of advanced laryngeal SCC is higher than that of early-stage cancer (Z=−3.006, P<0.05). The positive rate of lymph-metastasis group and that of non-lymph-metastasis group was 82.1 and 47.7%, respectively, the expression difference was statistically significant (Z=−4.129, P<0.05). The proportions of positive staining of the smoking group and that of the non-smoking group were 67.6 and 39.6%, respectively, the expression difference was significantly different (Z=−3.190, P<0.05).

Differences in Ang-2 expression according to gender, age, primary tumor site or drinking habits were not statistically significant (Table III).

### Association between cyclin D1 expression and laryngeal SCC clinical pathological factors

The proportions of positive staining of cyclin D1 in the well, moderately and poorly differentiated laryngeal SCC group tissues were 50, 66.7 and 100%, respectively. Interclass comparisons of the expression in the well and moderately differentiated groups and well and poorly differentiated groups were statistically significant (Z=−2.814, P<0.05 and Z=−4.087, P<0.05). No statistically significant difference of expression was observed between the moderately and poorly differentiated groups. The proportions of positive staining of laryngeal SCC in the T1+T2 and T3+T4 TNM stages were 52.0 and 78.0%, respectively. The positive rate of cyclin D1 increased with later pathological stage. The expression of advanced laryngeal SCC was higher than that of early-stage laryngeal SCC (Z=−3.182, P<0.05). The proportions of positive staining of the lymph-metastasis and non-lymph-metastasis groups were 82.1 and 54.5%, respectively; the expression difference was statistically significant (Z=−4.211, P<0.05). The proportions of positive staining of the smoking and non-smoking groups were 70.6 and 47.9%, respectively; the expression difference was statistically significant (Z=−2.518, P<0.05).

The expression of cyclin D1 was not significantly associated with gender, age, primary tumor site or drinking habits (Table IV).

### Correlation analysis of Ang-2 and cyclin D1 expression in laryngeal SCC

In this study, all cases were stained for cyclin D1 and Ang-2 simultaneously. There were 28 cases negative for both and 48 cases positive for both Ang-2 and cyclin D1. Spearman’s rank correlation analysis results demonstrated a positive correlation between cyclin D1 and Ang-2 expression in laryngeal SCC (r=0.5042; P<0.05; Table V).

### One-way analysis of clinically pathological parameters and laryngeal SCC patient survival time

Using log-rank one-way analysis, we demonstrated that laryngeal SCC prognosis is associated with smoking, TNM stage, lymph metastasis, tumor classification, tumor location, cyclin D1 and Ang-2, but is not correlated with age, gender or drinking habit (Table VI).

### Survival analysis

**Figure 9.**
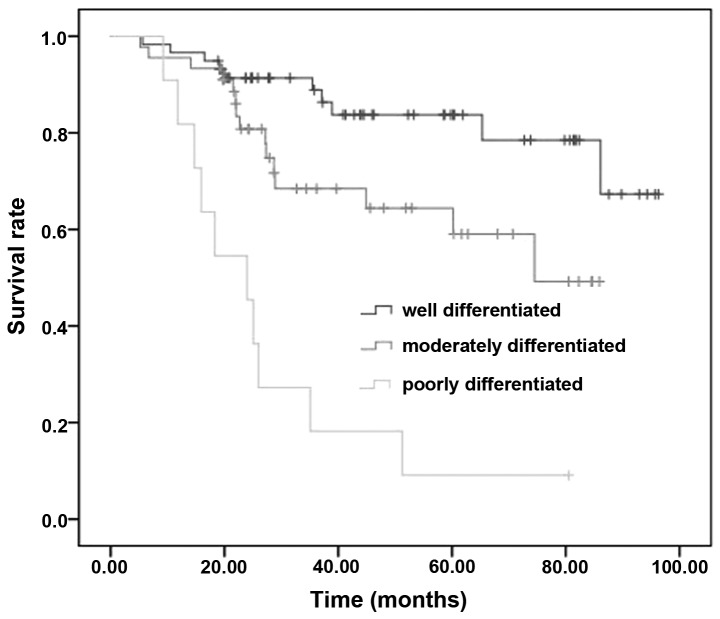
Survival rate of patients with laryngeal SCC in the well, moderately and poorly differentiated groups. SCC, squamous cell carcinoma.

**Figure 10.**
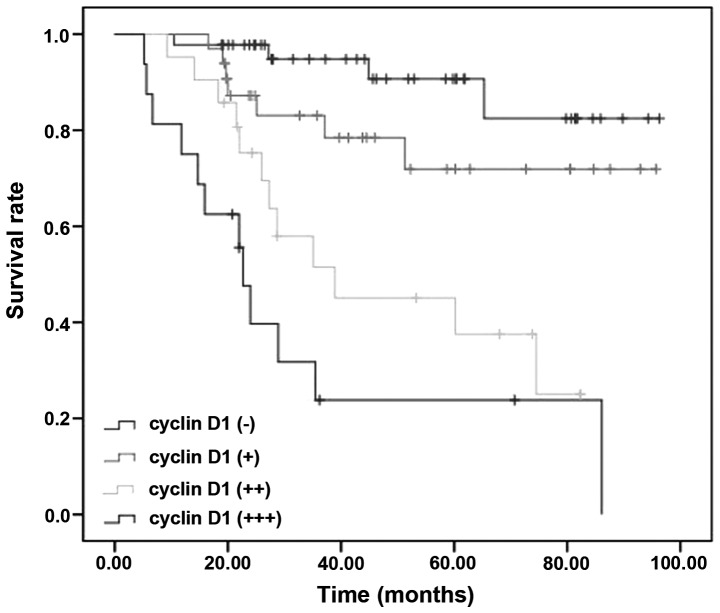
Survival rate of patients in cyclin D1-/+/++/+++ groups.

Using multi-factor Cox proportional hazards regression models to analyze TNM stage, lymph metastasis, tumor classification, tumor location, cyclin D1 and Ang-2, the tumor classification and cyclinD1 are associated with the survival rate (Figs. 9 and 10). The survival rate of well differentiated, moderately differentiated and poorly differentiated groups was 78.3, 66.7 and 0.91%, respectively. The survival rate of of cyclin D1 −/+/++/+++ groups was 88.9, 76.5, 42.9 and 25.0%, respectively. The tumor classification and cyclin D1 each were able to act as independent prognostic factors.

## Discussion

Similar to the majority of solid tumors, laryngeal SCC induction and development is subject to various factors, including modulation of tumor angiogenesis and the cell cycle. As a member of the angiopoietins, Ang-2 exerts a regulatory effect on angiogenesis (7). Cyclin D1 is classified as an oncogene. Once cyclin D1 mutates or expresses excessively during the process of cell proliferation, cyclin D1 promotes the induction and development of a tumor (8).

The Ang-2 protein is composed of 496 amino-acids with a molecular weight of 70 kDa, the gene for which is located at chromosome 8p23.1. The protein comprises a signal peptide, amino-end α-spiral hinge and carboxy-end fibrous protein sample regions (9). Ang-2 mainly exists in the form of a homodimer and is able to combine with vascular endothelium Tie-2 without causing phosphorylation of the receptor. However, Ang-2 is able to competitively block the effect of Ang-1, relax endothelial cells and perivascular sertoli cells, and accelerate vascular bed degeneration and extravascular substrate degradation, which renders the vascular net unstable, thus providing favorable conditions for endothelial cell division and new blood vessel reconstruction (7).

The study by Yan *et al* (10) on 64 cases of colorectal cancer has observed that, as the differentiation decreases, the expression rate of Ang-2 increases, which indicates that Ang-2 propelled the deterioration of the tumor. A study by Chen *et al* (11) on 51 cases of hepatocellular carcinoma suggested that Ang-2 is a marker of hepatocellular carcinoma. Research by Wang *et al* (12) on 50 cases of cervical cancer has demonstrated that as the differentiation decreases, the expression rate of Ang-2 tended to increase. In addition, the microvessel density rose markedly, which indicated the involvement of Ang-2 in hepatocellular carcinoma development (13). A study of 335 cases of non-small cell lung cancer by Anderson *et al* (14) studied the correlation between the expression of Ang-2 and prognosis, which showed that Ang-2 was a signal of tumor prognosis. Hashizume *et al* (15) suggest that Ang-2 may slow down the growth of tumors and therefore accelerate tumor cell death.

The results of the present study demonstrated that Ang-2 expression in laryngeal SCC specimens was markedly higher than that in atypical hyperplasia and vocal cord polyp tissues (P<0.05). Ang-2 was mainly located in the cytoplasm of laryngeal SCC cells. Therefore, we suggest that tumor cells generate Ang-2, which functions through autocrine and paracrine systems. Ang-2 expression in cancer tissues was markedly higher than that in normal laryngeal mucosal tissues. As the differentiation decreased, the expression of Ang-2 increased. We hypothesize that Ang-2 induces the split and shift of endothelial cells, permitting new vessels to grow by budding to promote the occurrence and development of a tumor. This study also showed that as the clinical stage advanced, Ang-2 expression increased. The expression of Ang-2 in the moderately and poorly differentiated groups was higher than that in the well differentiated group. Expression was higher in the lymph-metastasis group than in the non-lymph-metastasis group, which indicates that Ang-2 may promote laryngeal SCC infiltration and lymph metastasis.

Cyclin D1, the key protein of the G1 stage during cell proliferation, may interact with multiple proteins and transition the cell into the S stage. Therefore, it is considered a significantly positive regulator in the G1/S transition in the cell cycle (16). When the level of cyclin D1 increases, the G1/S stage shortens, with cell proliferation and canceration. There are various ways for the cyclin D1 gene to mutate within tumors, including gene amplification, chromosome translocation and inversion, among them gene amplification is the most common. In a study by Marsit *et al* (17), 698 cases of head and neck SCC underwent immunohistochemical testing. The results showed that, cyclin D1 was expressed in head and neck SCC tissues. Cyclin D1 was highly expressed in T3+T4 stages, but was rare in T1+T2. This indicates that cyclin D1 is relevant to tumor stage and may be regarded as a potential prognostic signal of head and neck SCC.

Our results have demonstrated that the expression of cyclin D1 in laryngeal SCC tissues is markedly higher than that in atypical hyperplasia and vocal cord polyp tissues (P<0.05). As the differentiation decreased, the expression of cyclin D1 increased, which indicated that the upregulated expression of cyclin D1 was associated with the degree of malignancy of laryngeal SCC. Solid tumor growth, proliferation and metastasis require new vessel support. At present, there are few studies concerning the correlation of cyclin D1 and Ang-2 expression with tumors. The present study showed that positive correlation exists between cyclin D1 and Ang-2 expression in laryngeal SCC, which indicates that both exert a synergic effect on laryngeal SCC occurrence, development and metastasis. We conducted analysis of 116 laryngeal SCC patients through log-rank one-way method and observed that laryngeal SCC prognosis is associated with smoking, TNM stage, lymph metastasis, tumor classification, tumor location, cyclin D1 and Ang-2, and is not associated with age, gender or drinking habit (Table 6). Using multi-factor Cox proportional hazards regression models to analyze TNM stage, lymph metastasis, tumor classification, tumor location, cyclin D1 and Ang-2, it was demonstrated that the tumor classification and cyclin D1 are associated with the survival rate (Figs. 9 and 10). The tumor classification and cyclin D1 were able to act as independent prognostic factors. Log-rank one-way method and multi-factor Cox proportional hazards regression models for tumor classification identified cyclin D1 as the independent factor affecting patient survival time (Figs. 9 and 10). Furthermore, this study did not observe any association between cyclin D1 expression and patient gender, age or drinking habit.

## Figures and Tables

**Table I.** Ang-2 expression in vocal cord polyp, atypical hyperplasia and laryngeal SCC tissues.

Histopathological type	n	Ang-2 expression		
		Negative	Positive	Positive rate (%)
Vocal cord polyp	5	5	0	0.0
Atypical hyperplasia	5	5	0	0.0
Laryngeal SCC				
Well differentiated	60	36	24	40.0
Moderately differentiated	45	15	30	66.7
Poorly differentiated	11	0	11	100.0

Ang-2, angiopoietin-2; SCC, squamous cell carcinoma.

**Table II.** Cyclin D1 expression in vocal cord polyp, atypical hyperplasia and laryngeal SCC tissues.

Histopathological type	n	Cyclin D1 expression		
		Negative	Positive	Positive rate (%)
Vocal cord polyp	5	5	0	0.0
Atypical hyperplasia	5	5	0	0.0
Laryngeal SCC				
Well differentiated	60	30	30	50.0
Moderately differentiated	45	15	30	66.7
Poorly differentiated	11	0	11	100.0

SCC, squamous cell carcinoma.

**Table III.** Expression of Ang-2 and clinicopathologic characteristics of the patients with laryngeal SCC.

Characteristics	n	Ang-2 expression				Z	P-value
		−	+	++	+++		
Gender							
Male	107	47	22	20	18	−0.038	0.9695
Female	9	4	2	1	2		
Age (years)							
≤60	43	18	10	8	7	0.127	0.8990
>60	73	33	14	13	13		
Smoking							
Yes	68	22	16	13	17	−3.190	0.0014
No	48	29	8	8	3		
Drinking							
Yes	49	26	8	7	8	1.350	0.1771
No	67	25	16	14	12		
TNM staging							
T1+T2	75	40	16	9	10	−3.006	0.0026
T3+T4	41	11	8	12	10		
Lymph node metastasis							
Yes	28	5	5	7	11	−4.129	0.0000
No	88	46	19	14	9		
Tumor differentiation							
Well	60	36	14	5	5	−3.258	0.0011
Moderately	45	15	8	12	10	−4.360	0.0000
Poorly	11	0	2	4	5	−2.274	0.0230
Primary tumor site							
Supraglottic	28	12	7	2	7	0.860	0.390
Glottic	73	36	11	14	12		
Subglottic	15	3	6	5	1		

Ang-2, angiopoietin-2; SCC, squamous cell carcinoma.

**Table IV.** Expression of cyclin D1 and clinicopathologic characteristics of the patients with laryngeal SCC.

Characteristics	(n)	cyclin D1 expression				Z	P-value
		−	+	++	+++		
Gender							
Male	107	42	33	17	15	−0.796	0.4260
Female	9	3	1	4	1		
Age (years)							
≤60	43	15	10	11	7	−0.045	0.9641
>60	73	30	19	11	13		
Smoking							
Yes	68	20	21	16	11	−2.518	0.0118
No	48	25	13	5	5		
Drinking							
Yes	49	18	17	6	8	−0.106	0.9159
No	67	27	17	15	8		
TNM staging							
T1+T2	75	36	22	10	7	−3.182	0.0015
T3+T4	41	9	12	11	9		
Lymph node metastasis							
Yes	28	5	5	9	9	−4.211	0.0000
No	88	40	29	12	7		
Tumor differentiation							
Well	60	30	22	4	4	−2.814	0.0049
Moderately	45	15	9	13	8	−4.087	0.0000
Poorly	11	0	3	4	4	−2.071	0.0384
Primary tumor site							
Supraglottic	28	8	9	6	5	0.460	0.640
Glottic	73	33	21	13	6		
Subglottic	15	4	4	2	5		

SCC, squamous cell carcinoma.

**Table V.** Correlation analysis of Ang-2 and cyclin D1 expression in laryngeal SCC.

Cyclin D1 expression	n	Ang-2 expression			
		−	+	++	+++
−	45	28	8	8	1
+	34	18	10	4	2
++	21	4	4	5	8
+++	16	1	2	4	9
Total	116	51	24	21	20

Ang-2, angiopoietin-2; SCC, squamous cell carcinoma.

**Table VI.** One-way analysis of clinically pathological parameters and laryngeal SCC patient survival time.

Parameter	Group	χ^2^	P-value
Gender	Male/female	0.005	0.945
Age (years)	≤60/>60	2.072	0.150
Smoking	Yes/no	4.598	0.032
Drinking	Yes/no	0.333	0.564
TNM staging	T1+T2/T3+T4	6.026	0.014
Lymph node metastasis	Yes/no	16.744	0.000
Tumor differentiation	Well/moderately/poorly	34.527	0.000
Primary tumor site	Supraglottic/glottic/subglottic	8.456	0.015
Cyclin D1	(−)/(+)/(++)/(+++)	42.220	0.000
Ang-2	(−)/(+)/(++)/(+++)	22.393	0.000

SCC, squamous cell carcinoma.
